# Spontaneous symmetry breaking propulsion of chemically coated magnetic microparticles

**DOI:** 10.1038/s41598-022-21725-z

**Published:** 2022-10-21

**Authors:** Louis William Rogowski, Min Jun Kim

**Affiliations:** 1 Applied Research Associates, Albuquerque, NM 87110 USA; 2 Department of Mechanical Engineering, Southern Methodist University, Dallas, TX 75275 USA

**Keywords:** Medical research, Nanoscience and technology

## Abstract

Chemically coated micro/nanoparticles are often used in medicine to enhance drug delivery and increase drug up-take into specific areas of the body. Using a recently discovered spontaneous symmetry breaking propulsion mechanism, we demonstrate that chemically coated microparticles can swim through mucus solution under precise navigation and that certain functionalizations can dynamically change propulsion behavior. For this investigation biotin, Bitotin-PEG3-amine, and biotin chitosan were chemically functionalized onto the surfaces of magnetic microparticles using an avidin–biotin complex. These chemicals were chosen because they are used prolifically in drug delivery applications, with PEG and chitosan having well known mucoadhesive effects. Coated microparticles were then suspended in mucus synthesized from porcine stomach mucins and propelled using rotating magnetic fields. The relationship between different chemical coatings, microparticle velocity, and controllability were thoroughly explored and discussed. Results indicate that the biotinylated surface coatings altered the propulsion behavior of microparticles, with performance differences interlinked to both magnetic field properties and localized mucus properties. Precisely controlled drug carrying microparticles are envisioned to help supplant traditional drug delivery methods and enhance existing medical techniques utilizing micro/nanoparticles.

## Introduction

Microrobotics technology has enormous potential to create a paradigm shift in medical treatment, enabling targeted drug delivery, minimally invasive surgery, and contrast enhancement for medical imaging. Specific applications include conserving rare therapeutics through precise delivery, de-clogging arteries, and brain surgery. To circumvent low Reynolds number physics^1^, a variety of microrobots have been developed to produce non-reciprocal motion, including helix based microrobots that take advantage of chiral geometry to propel^2^ and flexible microrobots that deform their bodies to create translational motion^3,4^. Additionally, Janus particles have been developed to propel through bulk fluid using chemical decomposition^5^ and self-generated thermal gradients^6,7^. These propulsion methods are effective and situationally necessary, but come with the cost of complexity and often require expensive fabrication steps^8^. While drug delivery using these platforms has been studied^9–11^, there would be enormous benefit in converting existing micro/nanoparticles already used in medical treatments into fully navigable microrobots. In addition to helping develop new targeted therapeutic treatments, such an advance would bolster existing research into micro/nanoparticle applications, such as drug delivery^12^, hypothermia^13^, and magnetic resonance imaging^14^.

In support of this effort, spontaneous symmetry breaking propulsion was recently discovered to occur in non-Newtonian rod-climbing like fluids, allowing symmetric magnetic microparticles to propel along their rotation axis using a squeezing effect from fluid first and second normal stress differences^15^. Prior to this study, the simplest objects documented to achieve propulsion in non-Newtonian fluids were dumbbell swimmers and 3D printed scallops^16,17^. While spontaneous symmetry breaking is currently limited to a subset of non-Newtonian fluids with rod-climbing-like properties, these effects were demonstrated to occur within mucus fluids synthesized from biological porcine mucin^15^. Given the complexity and physiochemical interactions mucins can have in drug delivery, a follow up investigation into how surface chemical functionalization affects spontaneous symmetry breaking propulsion is necessary.

The original investigation was restricted to microparticles coated with a streptavidin chemical functionalization (avidin)^5^. Avidin is known to interact with biotin and create one of the strongest non-covalent bonds found in nature^18^, making it highly desirable in medicine and nano-technology applications^19^. For these reasons, many drug compounds often have biotin functional groups that allow them to attach to micro/nanoparticles or other delivery vehicles coated with avidin. Common drug treatments in the gastrointestinal tract often rely on mucoadhesive compounds, to both increase uptake of drug payloads and ensure correct localization of drug carrying nanoparticles^20^. This brings to question whether chemically coated microparticles swimming under spontaneous symmetry breaking can (1) swim effectively inside mucus fluids and (2) what velocity differences or interactions occur in between different coatings. Being able to navigate microparticles quickly and effectively would be paramount to transferring them to real world use applications where speed in drug deployment procedures would be critical for patient recovery. Unlike passive micro-/nanoparticles previously examined, propelling microparticles will be able to precisely navigate to target locations, and penetrate through complex fluids and tissue environments, without relying solely on diffusion properties.

This study investigates the effects commercially available biotinylated chemical coatings have on spontaneous symmetry breaking propulsion within a mucus solution. The chemical coatings selected for this investigation include the original avidin surface coating (Case 1-acts as a control), a biotin-avidin complex [Case 2], a biotinylated polyethylene glycol amine (Bitoin-PEG3-amine, BroadPharm) [Case 3], and biotin chitosan (CH-Bitoin-2k, HAworks) [Case 3]. The latter two compounds [Case 3, PEG and chitosan] have documented mucoadhesive effects and are often used in conjunction with medically tailored compounds to treat specific ailments^20^. The standalone biotin compound [Case 2] was selected to understand how the basic linker group behaved in comparison to its composite forms. Microparticles with 10 µm diameter and each chemical coating were suspended in a mucus solution and rotated at different magnetic field amplitudes and frequencies. The results reported here include a detailed analysis of microparticle velocity vs. frequency under different magnetic field properties, the propulsion of microparticles under closed-loop feedback control, the effects static field has on propulsion behavior, and a discussion on how chemical structure possibly contributes to microparticle propulsion. This research demonstrates for the first time that microparticles, propelled using spontaneous symmetry breaking, can have their propulsion velocities directly affected by chemical functionalizations and perform predictable navigation through mucus fluids.

## Experimental setup

### Coating of microparticles

Magnetic microparticles with 10 µm diameter (Spherotech, SVFM-100-4) were used throughout the experiments. These microparticles came prefabricated with a surface coating of streptavidin (also known as ‘avidin’), enabling the natural attachment of any biotinylated compounds along their surfaces. For the experiments presented in this paper four different surface coatings were explored including: streptavidin with no biotinylated compounds, streptavidin combined with biotin (B4501, Sigma Aldrich), streptavidin combined with biotinylated polyethylene glycol amine (Biotin-PEG3-amine, Broadpharm), and streptavidin combined with biotin chitosan (CH-Biotin-2k, HAworks, USA, also known as 'chitosan biotin' in literature). Avidin coated particles were explored thoroughly in previous work^5^ with the re-experiments performed here for comparative and validation purposes. The streptavidin–biotin coating was explored as a secondary control group to understand how a biotin surface coating by itself, without any complex molecules attached, could potentially impact propulsion behavior. The other two surface coatings (polyethylene glycol and chitosan) are heavily documented in literature to produce mucoadhesive effects that allow for enhanced drug delivery and uptake in sensitive areas of the body^20–26^.

**Figure 1:**
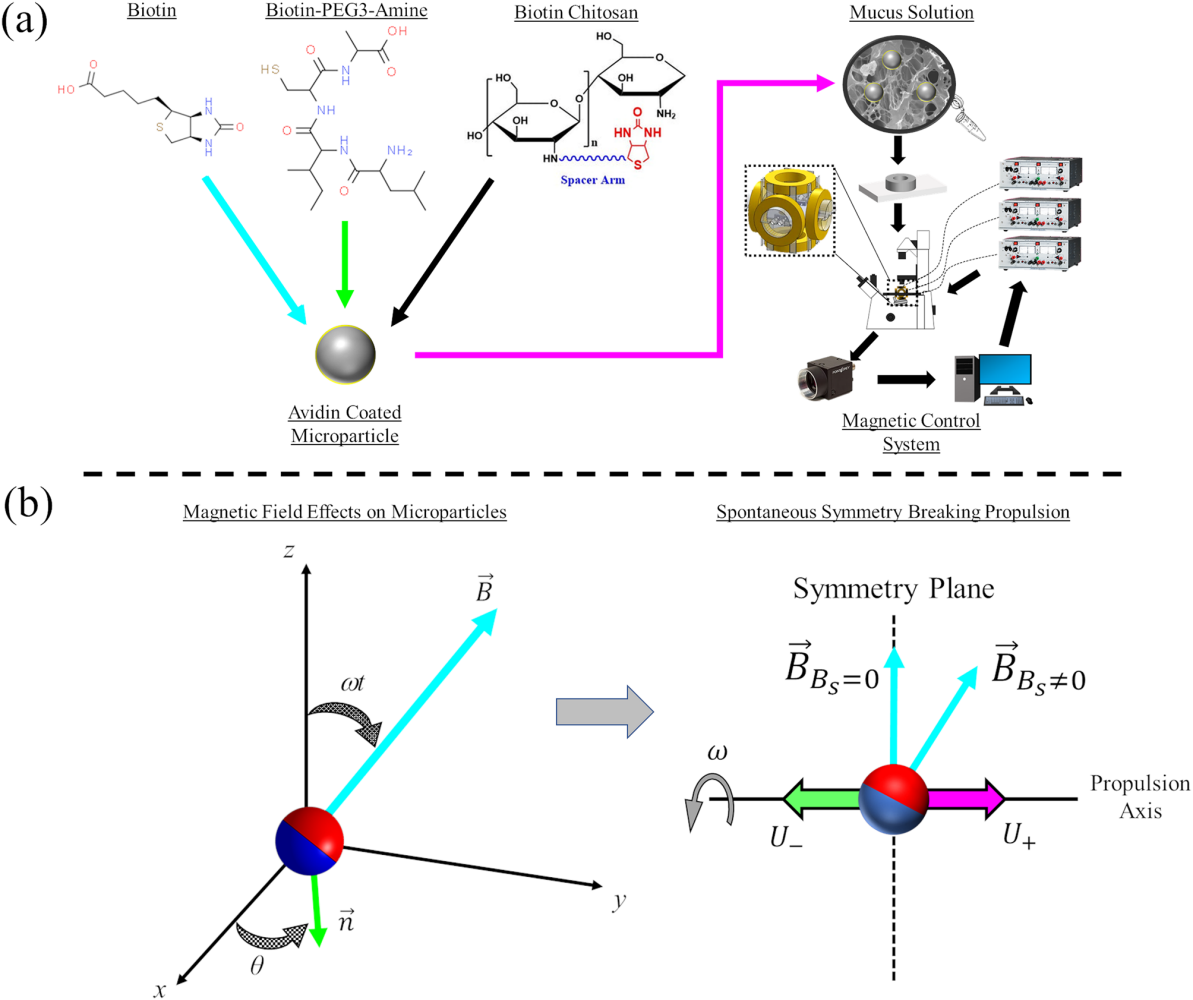
Overview of experimental setup and magnetic field interactions. (**a**) Avidin coated magnetic microparticles are functionalized with one of three compounds: biotin, Biotin-PEG3-amine, or biotin chitosan. Functionalized microparticles are suspended in a 4% mucin solution and loaded into a sample chamber which is placed in the middle of an approximate Helmholtz coil system. Programmable power supplies and camera visualization are used to navigate microparticles through the mucus with rotating magnetic fields. Chemical structures were extracted from Chemspyder and HAworks. (**b**) Magnetic fields produced from the Helmholtz coil system and their relationship to Eqs. (1–3). When torqued by a magnetic field, a microparticle in a rod-climbing-like fluid will propel along a propulsion axis perpendicular to its plane of symmetry. Two propulsion states can be achieved (*U*_+_,*U*_−_) randomly when no static field is applied (*B*_s_ = 0). Either propulsion state can be selected at will when a non-zero static field is applied (*B*_s_ ≠ 0). Red and blue hemispheres represent magnetic dipoles.

A 0.022% solution of biotin was created by mixing 2.2 mg of biotin (Sigma Aldrich, B4501) with 1 ml of deionized water; heating was used to help dissolve the biotin as well as mixing with a vortexing machine. Higher concentrations of biotin could not be explored due to solubility limitations of the biotin used (only 22 mg/100 ml). A 1% solution of Biotin-PEG3-amine was created by mixing 10 mg of Biotin-PEG3-amine with 1 ml of deionized water, with the same vortexing machine used to mix the solution. A 1% biotin chitosan solution was created by mixing 300 µl of deionized water with 3 mg of CH-Bitoin-2k; a vortexing machine was used again to thoroughly mix the solution. Microparticles at 1% concentration were added in 1 µl volume to an empty 1.5 ml centrifuge tube, with one of the three solutions selected and added to the tube in a 2 µl volume. The combined solution was allowed to sit for about a minute to allow for interaction between the biotinylated compounds and the surface of the avidin particles. Next, 1 ml of 4% mucin solution was added to the centrifuge tube and vortexed to ensure the particles were dispersed throughout the fluid medium. A strong permanent magnet (0.12 Tesla) was then placed next to the tube for 15 s to ensure that all microparticles were sufficiently magnetized before experimentation. A sample chamber was prepared by cutting a circular hole into a polydimethylsiloxane (PDMS) film that was 1 mm in height. The PDMS was then placed on a No. 1.5 cover slip and 30 µl of the mucus particle solution was added to the chamber; the chamber was then sealed with another No. 1.5 cover slip and any excess fluid leaks were removed with tissue paper. The chamber was then placed inside the magnetic control system and allowed to settle for several minutes before experimentation began; this was done to eliminate any internal flows. Microparticles remained in suspension and did not immediately sink to the bottom of the sample chamber due to the viscosity of the mucus solution. Microparticles were examined far from the boundaries of the sample chamber and only in bulk fluid. An overview of the coating process can be seen in Fig. 1a.

### Magnetic field controller

A custom-built approximate Helmholtz coil magnetic control system was utilized throughout the experiments to produce fields in three dimensions (3D). The six coils were produced using AWG-25 magnetic wire and had 600 turns each. The coils were separated by a 64.5 mm gap between each coil pair. Three programmable bipolar power supplies (KEPCO BOP-20-5 models) were used to provide a maximum of 20 Volts and 5 amps to each coil pair (one power supply per coil pair). A custom-built LabVIEW program was used to control the power supplies and produce desired rotating and static magnetic fields. Signals were sent to the programmable power supplies using DAQ boards (National Instruments) and were limited to only producing up to 10V output signals. The KEPCO power supplies acted as amplifiers and doubled the input signal provided (20V max). This 10V output signal limited the available frequencies microparticles could rotate at; this is due to the fact the amplitude of the rotational magnetic field is coupled with rotation frequency (explained below). This same magnetic control system was used extensively in previous work and has been well characterized in literature^15,27^. An overview of the magnetic control system can be found in Fig. 1a.

Rotating magnetic fields were generated using equations,

1 $$\overrightarrow{B}= \left(\begin{array}{l}-{B}_{s} \, \mathrm{cos}\,\theta +{B}_{r} \, \mathrm{sin}\,\theta\, \mathrm{cos}\,\omega t\\ {B}_{s} \, \mathrm{sin}\,\theta +{B}_{r} \, \mathrm{cos}\,\theta \, \mathrm{cos}\,\omega t\\ {B}_{r} \, \mathrm{sin}\,\omega t\end{array}\right),$$

2 $${B}_{r}=\beta f,$$

3 $$\overrightarrow{n}= \left(\begin{array}{ccc}-\cos \, \theta & \sin \, \theta & 0\end{array}\right),$$

where ${B}_{s}, {B}_{r}, \theta ,\omega , t$ are the static magnetic field amplitude, rotational magnetic field amplitude, heading angle in the *x–y* plane, the rotation of the field in radians per second, and the time in seconds, respectively. The rotational magnetic field amplitude was defined to be a function of frequency ($f$) in Hz, where $\omega =2\pi f$ and the magnetic field scaling factor $\beta$ is a ratio between frequency and the amplitude of ${B}_{r}$. This scaling factor is necessary to limit the occurrence of ‘step-out’ frequency, whereby the magnetic microparticle is rotating asynchronously from the magnetic field due to resistance from fluid viscosity^28^. The heading vector $\overrightarrow{n}$ shows the propulsion direction of the microparticle.

The spontaneous symmetry breaking propulsion mechanism occurs when magnetic microparticles are rotated within rod-climbing-like fluids, with propulsion velocity being heavily influenced by magnetic field properties^15^. As a microparticle rotates, first and second normal stress differences within the fluid create a squeezing effect along the propulsion axis perpendicular to the microparticle’s symmetry plane (Fig. 1b). This squeezing effect enables magnetic microparticles to experience one of two equal and opposite propulsion states (${U}_{+}, {U}_{-}$) that are randomly selected when a static field isn’t applied along $\theta$(${B}_{s}=0$). A single propulsion state can be repeatably selected once a super-imposed static magnetic field is applied (${B}_{s}\ne 0$), with the sign and magnitude of the static field determining which propulsion state is activated. The static field ‘tilts’ the magnetic dipoles of the microparticle and changes how the particle rotates about the propulsion axis; when ${B}_{s}=0$ the dipoles are perpendicular to the propulsion axis. An overview of the magnetic field interactions with magnetic microparticles can be found in Fig. 1b with the supplementary material of previous work explaining this tilting behavior in depth^15^.

For certain experiments, a proportional feedback controller was used to navigate microparticles to select points within the field of view. The feedback controller is defined as:

4 $$\dot{\theta }=k{\alpha }_{d},$$

5 $${\alpha }_{d}=\psi -\theta ,$$

where $\dot{\theta }$ is the time derivative of the heading angle in the *x–y* plane, $k$ is a proportional constant, $\psi$ is the desired heading angle of the microparticle, and ${\alpha }_{d}$ is the difference between the desired heading angle and the current heading angle of the controller. Coordinate positional data is obtained using real-time image processing to extract the microparticle's centroid and process fed into the controller. Target points for the microparticle are manually placed by the user and changed once the micropraticle reaches close to the desired user-determined destination. For all experiments, the proportional constant $k$ was set to 5, ensuring the controller reached steady state quickly, and the sample rate was fixed at 30 Hz.

## Results and discussion

### Characterization of mucus solution

Mucus solution was first synthesized by mixing mucin from a porcine stomach (Sigma Aldrich, M2378) with deionized water. For this study, only mucus solution in a 4% concentration was explored. Six grams of mucin was first mixed with 150 ml of deionized water for 30 min while heated to 60 °C. The solution was then transferred to three 50 ml centrifuge tubes and centrifuged at 1200 relative centrifugal force (rcf) for 10 min. After centrifugation, the supernatant of the tubes was transferred to new tubes while any excess undissolved mucus aggregates at the bottom of the tube were discarded. The mucus was then stored in a laboratory refrigerator at 4 °C until needed for experiments.

**Figure 2:**
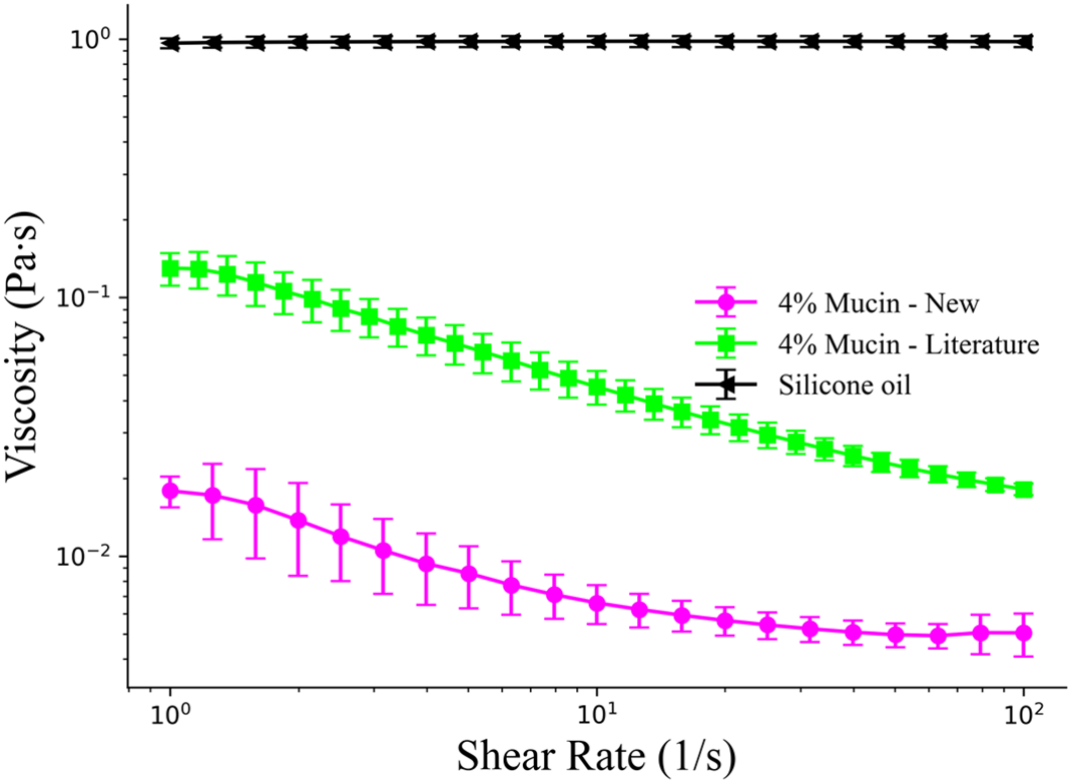
Viscosity vs. shear rate curves for different fluids. The data points in ‘4% Mucin–Literature’ were obtained from literature, while ‘4% Mucin–New’ contains data obtained from recharacterization. Silicone oil was used to demonstrate behavior of a Newtonian fluid and verify rheometer calibration. Error bars represent standard error over a minimum of three trials. Lines between points are a visual aide and do not represent interpolations.

Mucus solution prepared in this fashion, and regular biological mucus, have been characterized extensively in literature using rheology^15,29–31^. While these synthesized mucus solutions are missing a great deal of components, such as proteins, lipids, salts, DNA, cells, and cellular debris, the mucin glycoprotein accounts for the majority of mucus viscoelastic properties^29,32^. Before the introduction of coated microparticles, mucin rheology was briefly re-examined for comparative and validation purposes. A Discovery Hybrid Rheometer (DHR-3, TA Instruments) was used in combination with a 40 mm 4° cone geometry to acquire viscosity data. An incremental shear rate from 1 to 100 (1/s) with 30 s averaging time was used for each data point, with 10 data points acquired per logarithmic decade. Presented in Fig. 2 is the viscosity vs. shear rate curves for ‘4% Mucin—New’ synthesized during this study along with data that was previously reported in literature (labeled as ‘4% Mucin—Literature’)^15^. When compared with previous studies^15^, there was a significant drop in overall viscosity for the mucus solution formulated in this study. To verify the DHR-3 was correctly calibrated, silicone oil with a constant of viscosity of 1 Pa s was analyzed as a control and had values within 5% of the expected value (Fig. 2). As far as can be determined, the discrepancy between the mucus used here and previous experiments primarily comes from different batches of mucin provided by Sigma Aldrich. Mucus is known to vary heavily between individuals^29^, so it is not unreasonable for mucus solutions made from different porcine sources (pig stomachs) to also have wide variance in fluid properties. It is also possible that stochastic variations during the centrifugation step may also have led to this reduction in overall viscosity. Regardless, the mucus sample used for this study demonstrated non-Newtonian shear thinning effects similar to previously reported results^15^ and had viscosity close to the lower end of the correct range for biological mucus^29^. The presence of first and second normal stress differences was not re-examined here due to measurement difficulties discussed in the supplementary information of previous work^15^.

### Velocity profiles for different chemical coatings

**Figure 3:**
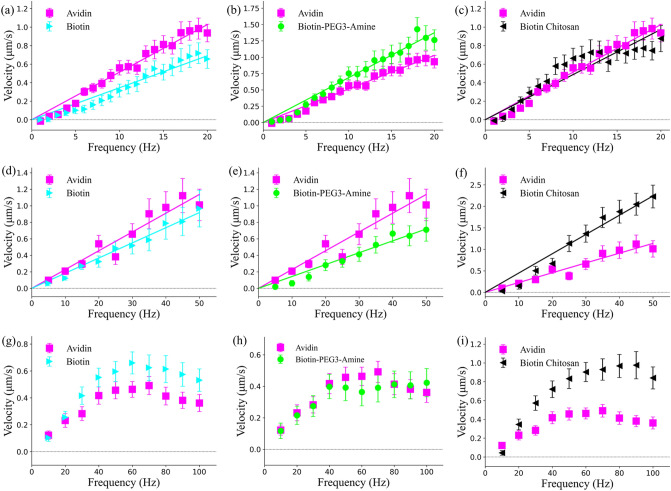
Velocity vs. frequency graphs for different chemical coatings, where all chemical coatings are compared against the avidin surface coating control case. (**a**–**c**) Velocity vs. frequency over a 1–19 Hz range, at 1 Hz increments, with a magnetic field scaling factor (β) of 0.5. (**d**–**f**) Velocity vs. frequency over a 5–50 Hz range, at 5 Hz increments, with a magnetic field scaling factor (β) of 0.175. (**a**–**c**) Velocity vs. frequency over a 10–100 Hz range, at 10 Hz increments, with a magnetic field scaling factor (β) of 0.1. Error bars represent standard error. The coefficients of determination for avidin, biotin, Biotin-PEG3-amine, and biotin chitosan in (**a**–**c**) are 0.97, 0.96, 0.97, and 0.78, respectively. The coefficients of determination for avidin, biotin, Biotin-PEG3-amine, and biotin chitosan in (**d**–**f**) are 0.93, 0.98, 0.96, and 0.96, respectively. Nine avidin particles, nine biotin particles, ten Biotin-PEG3-amine particles, and seven chitosan particles were examined in (**a**–**c**). Five avidin, five biotin, five Biotin-PEG3-amine, and five chitosan particles were examined in (**d**–**f**). Four avidin, five biotin, four Biotin-PEG3-amine, and five chitosan particles were examined in (**g**–**i**). All microparticles had at least 3 independent trials each. Solid lines represent linear fits starting at 0 Hz. Velocity was calculated only along the *x-*component, with *y-*component velocity being ignored.

Variable frequency experiments were conducted, using the magnetic field controller, to understand microparticle velocity behavior with different chemical coatings and magnetic field properties. For this set of experiments, microparticles were made to propel along the $$x$$-axis, with their velocity only being measured along this specific propulsion direction. Three different frequency ranges were explored (1–19 Hz, 5–50 Hz, and 10–100 Hz) with each having a different scaling factor ($$\beta$$) for the rotational magnetic field amplitude ($${B}_{r})$$; the $$\beta$$’s for each range of frequencies were fixed at 0.5, 0.175, and 0.1, respectively. These $$\beta$$’s were selected such that the power supplies could provide sufficient power to the coils over the necessary frequency range without hitting their 20V upper limit. Rotational frequency was iterated at 1 Hz, 5 Hz, and 10 Hz increments for each of the respective frequency ranges. Microparticles were tracked using centroid positional data obtained from image processing, with their velocities obtained by instantaneously calculating particle position between individual frames. Results of the three different frequency ranges are shown in Fig. 3, with each surface coating compared against the streptavidin (avidin) coated control group.

The effects of different surface coatings become immediately apparent in the first set of experiments under a 1–19 Hz frequency range (Fig. 3a–c, $\beta =0.5$). Biotin imparts a noticeable decrease in velocity in Fig. 3a while maintaining a high coefficient of determination with its linear fit. Biotin microparticles stay within a standard deviation of the avidin particles at low frequencies (expected), but the two curves diverge at around 6 Hz and become distinct. It was unexpected for biotin to have a negative impact on performance as scarce literature exists regarding biotin interactions with mucin. In contrast, the Biotin-PEG3-amine surface coating (Fig. 3b) imparts a positive effect on microparticle propulsion, both increasing the overall mean velocity at each frequency above 5 Hz and greatly exceeding the propulsion of avidin microparticles after 17 Hz. The biotin chitosan surface coating (Fig. 3c) did not have a significant impact on microparticle propulsion, being nearly equivalent in linear fitting and only having a slight performance increase in velocity at low frequencies. Both Biotin-PEG3-amine and biotin chitosan were expected to have positive effects on microparticle propulsion due to their mucoadhesive effects increasing mucus particle interactions; but only Biotin-PEG3-amine demonstrated this behavior.

To further understand these results, the magnetic field scaling factor was adjusted ($\beta =0.175)$ such that lower amplitude magnetic fields were used but higher rotational frequencies could be achieved from the power supplies. The results for each respective surface coating can be seen in Fig. 3d–f for a frequency range of 5–50 Hz with a $\beta$ of 0.175. Under the new frequency range, the biotin surface coating (Fig. 3d) was now indistinguishable from the avidin surface coating control group, having nearly identical linear fits. The Biotin-PEG3-amine group (Fig. 3e) now imparted a negative performance, being slower overall compared to the avidin control group. The biotin chitosan surface coating (Fig. 3f) now displayed a faster and distinct velocity performance compared to the avidin control group, being nearly more than twice as fast at the 50 Hz increment.

Finally, the scaling factor $\beta$ was reduced again to 0.1 and a frequency range of 10–100 Hz was investigated. At this frequency range the velocity behavior of all four microparticle coatings becomes non-linear (Fig. 3g–i). A small linear region exists between 10 and 50 Hz, but afterwards there is a diminishing velocity for all four surface coatings. This non-linear behavior is most likely caused by step out frequencies experienced by the microparticles, whereby the magnetic field is rotating asynchrony with the microparticle. The biotin surface coating (Fig. 3g) now outperforms the avidin coating, with the mean velocity at each point being above the standard deviation of the avidin microparticles. The Biotin-PEG3-amine group (Fig. 3h) is now indistinguishable from the avidin microparticles velocity curve. The last set of microparticles examined with a biotin chitosan coating (Fig. 3i) were significantly faster and more distinct from the avidin coated particles.

The effects of different coatings are extremely dependent on the magnetic field characteristics of the control system. With high amplitude magnetic fields and low frequency, biotin behaves poorly compared to the control case. However as magnetic field amplitude is decreased and frequency increased, biotin coated microparticles can be made equivalent too or better than avidin microparticles under the same conditions. The inverse of this was true for Biotin-PEG3-amine coated microparticles, where under low frequencies and high magnetic field amplitude they outperformed avidin microparticles, but under high frequencies and low magnetic field amplitude they behaved worse or identical to the control group. Biotin chitosan showed comparable behavior to biotin, by increasing in velocity and distinctiveness from avidin surface coatings as magnetic field amplitude decreased and frequency increased.

**Figure 4:**
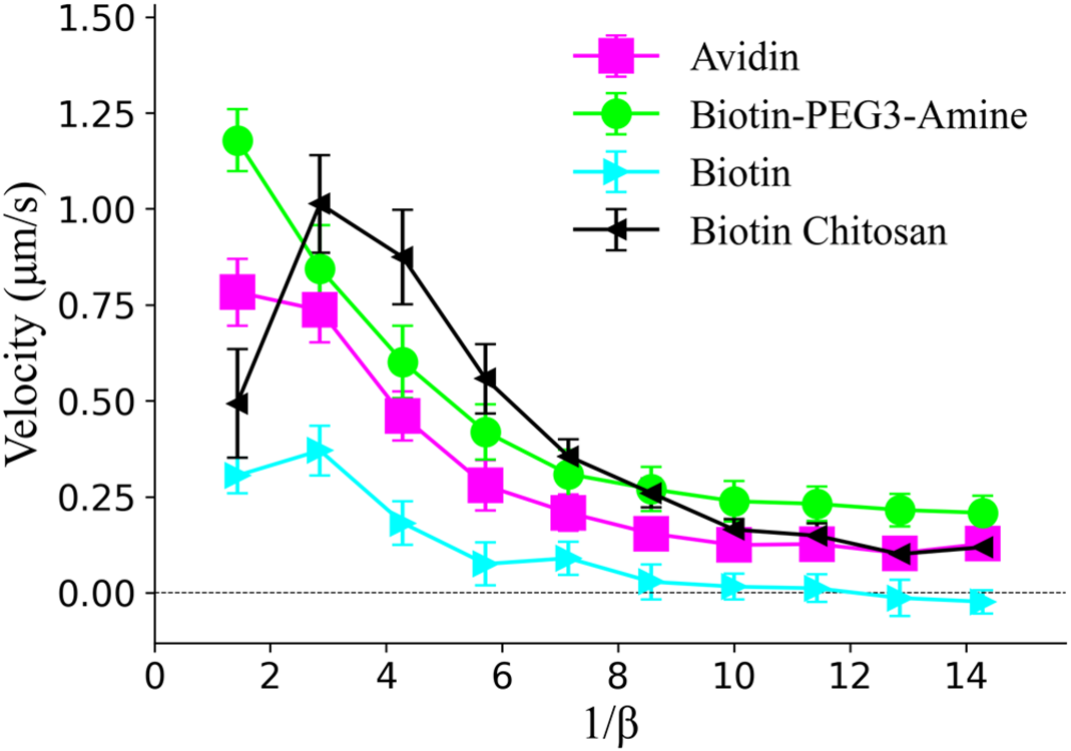
Velocity vs. magnetic field scaling factor for different chemical coatings. Rotation frequency of investigated particles was fixed at 14 Hz. Lines between points were added for visualization purposes and do not represent interpolations. Five avidin, three biotin, four Biotin-PEG3-amine, and four biotin chitosan particles were examined, with each having at least three trials each.

To better characterize this behavior, a separate experiment was conducted for each chemical coating, whereby rotation frequency of the microparticles were fixed at 14 Hz, but the magnetic field scaling factor was decreased with time. Figure 4 shows the results of this investigation, where 1/$\beta$ is plotted for clarity purposes along the *x-*axis. The velocity results plotted here are consistent with expectations outlined in Fig. 3a–c. Of the curves plotted, biotin chitosan has the most dynamic behavior, where at low 1/$\beta$ values, velocity is slow, then improves at moderate values, before finally decreasing again at higher 1/$\beta$ values. Biotin to a much lesser extent demonstrates a similar trend, with a velocity spike around 1/$\beta$ = 3. All particles behaved consistently from previous experiments. Higher frequencies could not be explored due to limitations in power supplies (see “Experimental setup” section).

### Analysis of user-defined trajectories

**Figure 5:**
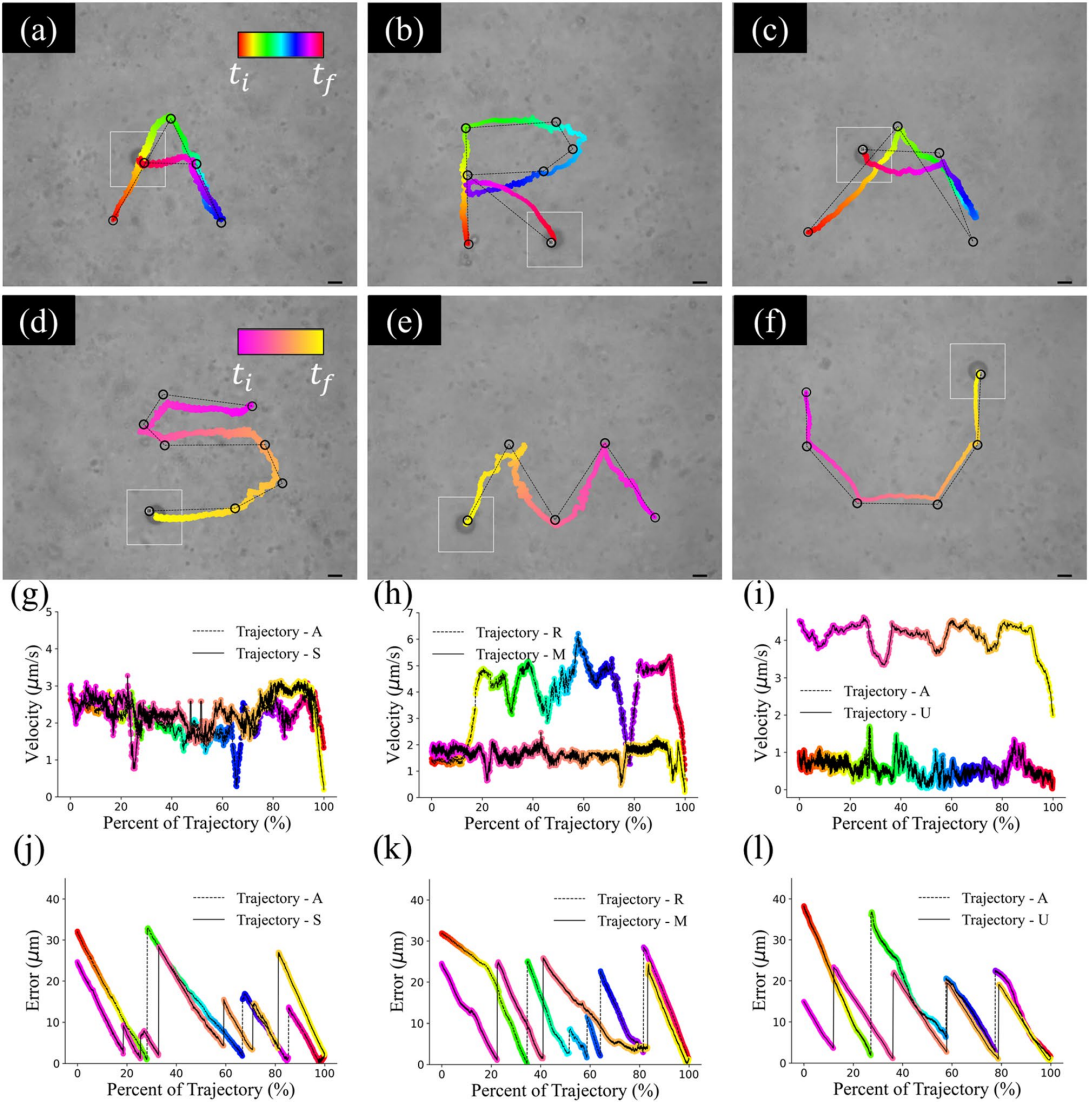
Trajectories of different chemically coated microparticles. Subfigures (**a**,**d**) represent a biotin coated microparticle, (**b**,**e**) represent biotin chitosan coated microparticles, and (**c**,**f**) represent Biotin-PEG3-amine coated microparticles. Subfigures (**g**–**i**) represent velocity graphs for the biotin, biotin chitosan, and Biotin-PEG3-amine microparticles, respectively. Subfigures (**j**–**l**) represent error in microns from each target location over time for each trajectory; sudden jumps in error indicate when a new target location was selected. Black circles represent target locations, while the dashed black line represents shortest path between individual target points. Black scale bar is 10 microns. Velocity graphs are shown as total velocity including *x- *and *y-*components. Trajectories in (**a**) and (**d**) are the same particle, while (**b**,**e**) and (**c**,**f**) were all performed by different microparticles. Timings for each trajectory were: (**a**) 62 s, (**b**) 95 s, (**c**) 269 s, (**d**) 71 s, (**e**) 74 s, and (**f**) 70 s. White box around each microparticle represents a bounding box for tracking the microparticle’s centroid. Velocity graphs were smoothed with a 60-point moving average and outliers beyond 3 × the mean of the velocity were set to the mean velocity value for each respective trajectory.

Microparticles were next tested to travel over user-defined trajectories under computer-controlled feedback control. A proportional feedback controller (see “Experimental setup”, “Magnetic field controller”) was used to navigate microparticles of biotin, biotin chitosan, and Biotin-PEG3-amine coatings through the mucus solution. Two trajectories were performed per microparticle coating, with their total velocities calculated at each frame captured along the trajectories. Microparticles were rotated at a constant frequency of 15 Hz with a $\beta$ of 0.5. Results of these feedback control experiments are presented in Fig. 5. All microparticles, invariant of their surface coatings, were able to complete each user-defined trajectory and produce their target shapes (‘A’, ‘R’, ‘A’ and ‘S’, ‘M’, ‘U’). In addition to the trajectories performed by the microparticles, the total velocity (*x–y* components), and error from target points, are also plotted. In Fig. 5a,d the same biotin coated microparticle was used for both experiments; when examining the velocity graph (Fig. 5g) we see that the propulsion velocity is consistent between the different trajectories. It is important to note here that the velocity for this biotin particle greatly exceeds the predicted value from Fig. 3a; this is primarily because the velocity shown here represents total velocity (including *x-* and *y*-components). Two different biotin chitosan particles were used to create the ‘R’ and ‘M’ trajectories in Fig. 5b,e. Examining Fig. 5h, we see that there are significant velocity differences between the particle in (b) and the particle in (e). Finally, when examining two different particles coated with Biotin-PEG3-amine (Fig. 5c,f,i), we find that the same discrepancy in velocity exists between individual microparticles, with some being well beyond the expected values measured in earlier velocity experiments. These velocity differences are speculated to be the result of localized mucus properties; this was observed in the SI of literature^15^ as micropraticle propulsion was measured as a function of distance from the boundaries of the sample chamber. As has been documented in literature^29^, mucus is heterogenous, with concentrated mucin glycoproteins being present randomly in solution. While only a few of these particles were examined for this set of experiments, we can see that in addition to chemical surface coatings, localized mucin concentrations also play a large role microparticle propulsion behavior. This does not invalidate the data presented in Fig. 3, as that was the aggregate data of multiple experiments and trials, but rather adds context that other factors could seriously influence individual microparticle propulsion. Finally, the error of each trajectory can be seen in Fig. 5j–l, where sudden spikes in error are the result of the target location being manually changed by the user to the next point in the trajectory. In all cases, the microparticles were able to either reach or come very close to their target destinations before a new destination was selected, with error mostly decreasing with time. Based on these results, we can conclude that microparticle control is achievable, regardless of surface coatings.

### Comparison of static magnetic field sweeps

**Figure 6:**
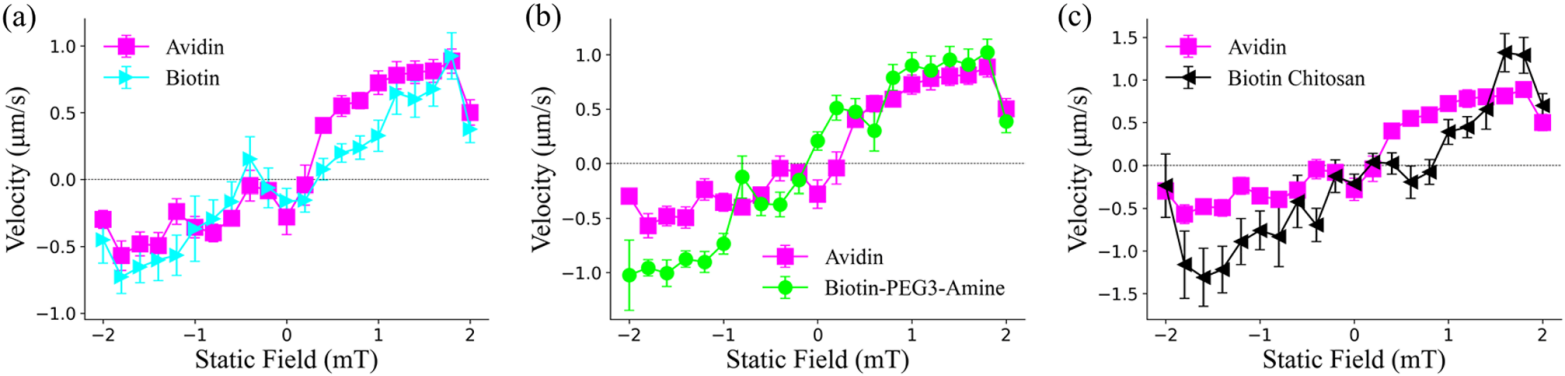
Static magnetic field sweeps for avidin, biotin, biotin chitosan, and Biotin-PEG3-amine coated microparticles. Static field was swept from − 2 to 2 mT at a 0.2 mT incrementation. Four avidin particles, five biotin particles, four chitosan particles, and four Biotin-PEG3-amine particles were used to produce the results in (**a**–**c**). At least three independent trials were performed per particle. Black horizontal line represents the *x*-intercept. Lines between points were added for visualization purposes and do not represent interpolations.

The final experiment performed was a static magnetic field sweep for each of the different coatings. The static magnetic field controls the tilt angle of the microparticle as it rotates and is directly responsible for switching between the two propulsion states^15^ (see “Experimental setup”, “Magnetic field controller”, Fig. 1b). As the static magnetic field is swept, the microparticle will eventually switch propulsion directions once a certain static field threshold is reached. In these experiments, the static field was swept from − 2 to 2 mT at 0.2 mT increments. The microparticles were rotated at a constant frequency of 15 Hz ($\beta =0.5, 15.87 \; \mathrm{mT})$ and made to propel along the $x$*-*axis. The results for experiments for each chemical coating are presented in Fig. 6. All microparticles, regardless of coating, behaved in much the same way and had similar velocity responses to static field incrementation. The only notable comments were that for the microparticles examined, both Biotin-PEG3-amine and biotin chitosan particles had increased velocity near the extremes of the static fields. Additionally, the static sweep profile of avidin microparticles varied considerably from previous work^15^, instead being similar in appearance to static sweep profiles obtained from microparticles propelling within polyacrylamide (PAA) solution. This discrepancy most likely comes from the difference in fluid properties discussed previously in the ‘Characterization of mucus solution’ section.

### Chemical functionalization and microparticle propulsion

The chemical coatings examined in our investigation were experimentally demonstrated to affect velocity behavior under spontaneous symmetry breaking. However, magnetic field differences and mucus properties also significantly influenced propulsion behavior. To better understand the interplay between these interactions, this section will attempt to highlight possible reasons for how the examined chemical functionalizations (Biotin-PEG3-amine, biotin chitosan, biotin, and avidin) could be modulating propulsion behavior. In our study, the only two compounds examined which possessed mucoadhesive interactions were polyethylene glycol (PEG) ^24,25^ and chitosan^21,23^, with the avidin and biotin-avidin coatings not having any meaningful literature to consult. The exact mechanism behind mucoadhesion is still greatly debated in literature but is believed to be a combination of electrostatic interactions, hydrogen bonding, van der Waals forces, surface tension, interpenetration, separation difficulty, surface roughness, and molecular weight (MW)^33^. While there are many investigations into these phenomena, we will discuss a handful of studies that can be related to our own findings.

The mucoadhesive interactions of PEG are documented to be the result of hydrogen bonding and electrostatic interactions, since PEG is naturally hydrophilic and possess a neutral charge^32^. However, other properties like molecular weight have also been found to alter mucoadhesion properties. Low density PEG (< 2 kDa) for instance was speculated to prevent polymer interpenetration with mucin glycoproteins, allowing enhanced penetration without mucoadhesive entanglement with mucin fibers^32^. This was quantified when large passive PEG coated nanoparticles of 100–500 nm diameter were observed in human cervical mucus and were measured to have a 4–6 × decrease in diffusivity than compared to suspension in a deionized water solution. Uncoated versions of these same diameter nanoparticles suspended in human mucus had diffusivity 2,400–40,000 × lower than when compared within a deionized water solution^32^. Additionally, some literature exists suggesting that PEGylated amine nanoparticles experienced negative surface charges when interacting with mucin glycoproteins, which varies depending on PEG chain length, with longer chains increasing transport capabilities^34^. Surface charge is believed to be neutral for Biotin-PEG3-amine in our experiments according to manufacturer documentation. The MW of Biotin-PEG3-amine used in our experiments, however, was only 418.6 Da, so it is likely that this surface coating could not create significant mucoadhesion during certain experiments, especially when rotation frequency was high, since there was less time available to adhere with surrounding mucin fibers. This could explain why under high torque and low rotation frequency, Biotin-PEG3-amine could achieve enhanced propulsion compared to just the avidin surface coating (Fig. 3b), since adhesion was more likely. Larger PEG chains (Biotin-PEG36-amine, BroadPharm, MW 1901.3 Da) may result in improved propulsion velocity under other magnetic field settings and will be explored in later experiments.

In contrast to PEG, researchers have reported that the driving factors behind mucoadhesion for chitosan are attributed to amino group counts, electrostatic forces, hydrogen bonding, and hydrophobia^35,36^. The interactions between chitosan and mucin glycoproteins are also influenced by molecular weight, concentration, ionic strength, environmental pH and a host of other environmentally sensitive and interdependent interactions^37^. These properties were leveraged in previous studies with nano/microrobots (NMRs), where negatively charged chitosan was surface coated to enhance penetration capabilities through electrostatic interactions^38,39^, since mucin glycoproteins are usually negatively charged at high pH^37^. While the NMRs explored in literature were catalytic Janus particles, they demonstrate that the surface coating is beneficial to active propulsion within in vivo environments and helped with aggregate swarms near target locations. Like Biotin-PEG3-amine, the biotin chitosan used in our study had a neutral charge, but also possessed a molecular weight of 2 kDa, which is significantly larger than the either Biotin-PEG3-amine (MW 418.6 Da) or biotin (MW of 244.31 Da) functionalizations examined. While molecular weight possibly explains high frequency and low magnetic field amplitude propulsion behavior (Fig. 3f,i), it does not explain the results of Fig. 3c, as this should have allowed more opportunities for mucoadhesion to occur. While speculative, it is possible that some unmodeled interactions occurring between rotating functionalized microparticles and mucin fibers cause a resonance that creates enhanced velocity, like what was seen within Fig. 4 for biotin chitosan and biotin coated microparticles at around 1/$\beta$ = 3.

As far as we are aware, there are no direct studies that quantify mucoadhesive interactions of avidin or biotin with mucin glycoproteins. This makes the behavior displayed by biotin coated microparticles difficult to explain. Streptavidin was the control group, but even that surface coating had a molecular weight of 66 kDa, so it would be strange for the addition of biotin (MW of 244.31 Da) to have a performance modification without some kind of explainable secondary interaction occurring. Based on our experiments the behavior of biotin correlates well with biotin chitosan, where higher frequencies and lower magnetic field amplitudes resulted in improved performance; the exact reasons for this similar behavior are difficult to determine at this time.

Even with available literature, it is difficult to conclude the driving factors behind the enhanced propulsion experienced by our coated microparticles. A lot of studies done investigating PEG and chitosan involved nanoparticles^32,40^, which are orders of magnitude smaller, and other literature involves experimental studies that cannot be directly related to our work^33^. Based on what we do know, it seems likely that molecular weight is a driving factor in mucin interactions, with electrostatic interactions also having some contributions. Given how new spontaneous symmetry breaking propulsion of microparticles is, however, we suspect that many more interactions remain unmodeled. Given the results of Figs. 3 and 4, relationships with non-linear shear thinning behavior also requires more investigative analysis.

## Conclusion

This investigation examined the propulsion effects of different chemical coatings on magnetic microparticles under a spontaneous symmetry breaking effect. Two mucoadhesive compounds, Biotin-PEG3-amine and biotin chitosan, were functionalized onto avidin coated microparticles and examined within a 4% mucin solution. Biotin, a non-mucoadhesive compound, and avidin coated microparticles already studied in literature, were also examined for comparative purposes. The results of the investigation determined that chemical compound functionalization can have moderate effects on spontaneous symmetry breaking propulsion, with other factors such as localized mucus properties and magnetic field characteristics (frequency and magnetic field scaling factor) being coupled with performance.

To summarize the results, under a low frequency range with a high magnetic scaling factor, microparticles coated with Biotin-PEG3-amine were faster than any of the other coatings examined. Biotin had a negative impact on propulsion and biotin chitosan had marginal effects under the same conditions. As scaling factor was reduced and rotational frequency was increased, biotin chitosan coated microparticles became significantly faster than the other coated microparticles, with Biotin-PEG3-amine imparting a negative performance, and biotin showing improved performance. As frequency was further increased and magnetic scaling factor reduced, the velocity profiles became non-linear due to step-out frequencies, with biotin chitosan being the fastest, followed by biotin, avidin, and Biotin-PEG3-amine. The effect of modulating magnetic scaling factor under a fixed frequency also clearly demonstrated that biotin chitosan coated microparticles had a dynamic velocity response that was only loosely experienced by the biotin-avidin coating. All coated microparticles could perform closed-loop feedback control using a proportional controller and could reach target locations easily. Examining microparticle velocity with each coating revealed two interesting features: (1) the same particle would have consistent velocity invariant of trajectory and (2) some microparticles experienced propulsion velocities outside of expectations; speculated to be the result of concentrated mucin glycoproteins acting as barriers to microparticle propulsion^29,30^. Finally, the static field sweep experiments demonstrated that microparticles can switch between spontaneous symmetry breaking propulsion modes, regardless of chemical coating, without significant velocity variations between coatings. It is difficult to conclude an exact relationship between chemical coatings and improved propulsion behavior; we speculate from literature that these interactions are the result of molecular weight and electrostatic force differences between different coatings. In addition, we believe that unexplored physiochemical interactions between active microparticles and mucin glycoproteins are heavily contributing to these results and make it difficult for effective comparisons with studies involving passive nanoparticles^32,40^.

In conclusion, the experiments performed here demonstrated that chemical functionalization could induce propulsion changes from spontaneous symmetry breaking propulsion and that the coated microparticles could propel in bulk mucus fluid. The work here focused on experiments using mucin extracted from porcine stomach, which acted as an allegory to expected behavior within biological mucus samples. Ultimately, mucus samples collected from mice and rats would be interesting to explore in future work, possessing trace cells and chemical compositions that were not factored into our results. However, a lot more work needs to be done to understand the interactions between spontaneous symmetry breaking and mucoadhesive properties from a physics standpoint. Coating these microparticles with an actual pharmaceutical compound and measuring uptake within in vivo environments using ‘swarms’ of microparticles, or examining cellular membrane interactions, are the next steps to validate this platform. Designing specialized surface coatings to generate specific propulsion properties will also provide new approaches to drug delivery strategies. It is hoped that this study will increase interest into micropraticle based propulsion mechanisms and help provide novel innovations to targeted drug delivery applications.

## Supplementary Information

Supplementary Information.

Supplementary Video 1.

## Data Availability

All data associated with this manuscript is available upon request from Dr. Louis William Rogowski.
