# Supplementary material for: Spontaneous symmetry breaking propulsion of chemically coated magnetic microparticles
Source: Sci Rep. 2022 Oct 21;12:17646. doi: 10.1038/s41598-022-21725-z (PMC9587300; doi:10.1038/s41598-022-21725-z)
Supplement: Supplementary file 1 — Supplementary Information. [file 41598_2022_21725_MOESM1_ESM.docx]

**Supplementary information guide: spontaneous symmetry breaking propulsion of chemically coated magnetic microparticles**

Louis William Rogowski^1,*^, Min Jun Kim^2,*^

^1^Applied Research Associates, Albuquerque, NM 87110

^2^Department of Mechanical Engineering, Southern Methodist University, Dallas, TX 75275

^*^Corresponding authors: [logrowski@ara.com](mailto:logrowski@ara.com) and mjkim@lyle.smu.edu

**Supplementary_Information.mp4**

This video demonstrates all major experiments discussed within the manuscript’s main text. Experiments are overlayed with scale bars, trajectory paths, and other experimental information to convey to viewers what’s going on in each experiment. Videos are sped up (3 -40 $\times$) to provide a concise overview of the select experiments.
